# Intracellular Uptake of Magnetic Nanocapsules with Ionic Chitosan Shells and Magnetically Triggered Cargo Release

**DOI:** 10.2147/NSA.S515639

**Published:** 2025-06-07

**Authors:** Elżbieta Gumieniczek-Chłopek, Joanna Odrobińska-Baliś, Adriana Gilarska, Gabriela Opiła, Manuel Ricardo Ibarra, Czesław Kapusta, Szczepan Zapotoczny

**Affiliations:** 1 Faculty of Physics and Applied Computer Science, AGH University of Krakow, Krakow, Poland; 2 Jerzy Haber Institute of Catalysis and Surface Chemistry, Polish Academy of Sciences, Krakow, Poland; 3 Faculty of Chemistry, Jagiellonian University, Krakow, Poland; 4 Instituto de Nanociencia Y Materiales de Aragón (INMA), CSIC-Universidad de Zaragoza, Laboratorio de Microscopías Avanzadas (LMA), Universidad de Zaragoza, Zaragoza, Spain

**Keywords:** magnetic nanoparticles, drug delivery systems, nanocarriers, nanomedicine, iron oxide nanoparticles, polymer capsules

## Abstract

**Introduction:**

Drug delivery systems typically need to be equipped with targeting moieties in order to be efficiently internalized by cells. Alternatively, magnetic nanoparticles (MNs) combined with active compounds may be driven by magnetic field to the site of action. Delivery of hydrophobic drugs using this approach is challenging as it would require coupling of MNs and hydrophobic environment within nanocarriers and triggering of the drug release.

**Methods:**

We propose an approach enabling a magnetically induced forced uptake of core-shell nanocapsules carrying hydrophobic actives together with hydrophobized MNs. Such capsules, formed in a facile emulsification process, are composed of amphiphilic cationic or anionic chitosan (shell) and oil-dispersible MNs (oil core). The capsules were characterized using DLS, cryo-TEM. They were loaded with a model fluorescent dye, Nile Red, and pulled into cells applying a static magnetic field. Then, they were treated with an alternating magnetic field to disrupt the capsules thanks to the action of MNs.

**Results:**

Cryo-TEM imaging confirmed the presence of MNs inside the capsules (d≈200 nm). Confocal microscopy imaging showed the efficient capsules’ intracellular uptake only after exposition to static magnetic field (some spontaneous uptake was observed for anionic capsules). Then, application of alternating magnetic fields induced rapture of the capsules inside the cells and release of the cargo.

**Discussion:**

This approach is very versatile as various lipophilic compounds could be encapsulated, then transported to desired tissues without active or passive targeting and kept there using static magnetic field, limiting undesired side effects of a therapy to the whole organism. The proposed capsules with MNs respond efficiently to magnetic field stimulation – they can be magnetically navigated into the cells and release their cargo after application of alternating magnetic field. This approach opens opportunities for controlled intracellular delivery of hydrophobic actives using easily applicable magnetic stimuli for both delivery and release.

## Introduction

Nanomedicine is a dynamically developing field that uses nanotechnology approaches to eliminate many diagnostic and therapeutic limitations by combining methodologies of various disciplines.^1^,^2^ Its development, stimulated, among others, by the increasing number of cases of neoplastic diseases,^3^ brings new therapeutic solutions based on targeted and controlled delivery of biologically active substances.^4^,^5^

The most common approach in targeted drug delivery is the application of ligand-based carriers, but it may be severely limited by nonspecific interactions of such carriers with complex media (like blood). Thus, even if such targeting appears effective during in vitro studies, common complications in clinical settings can occur.^6^ Various expression levels of receptors corresponding to specific ligands may further limit the impact of such ligand-based therapies.^7^ Furthermore, macromolecular ligand targeting is typically characterized by high costs, instability, and complexity of its characterization.^8^ Forced delivery might lead to the reduction or complete elimination of such problems by remote navigation of drug carriers followed by the controlled release that can both be realized by the application of a magnetic field.^9–13^ Iron oxide magnetic nanoparticles (MNs) can be utilized in such systems owing to their superparamagnetic properties and very promising results of in vivo studies.^14^ Magnetically navigated drug delivery systems may be formed by introducing such nanoparticles into larger structures (eg liposomes, micelles, capsules) serving as carriers, therefore they can respond to an external magnetic field.^15^ The applicability of dynamic microcapsules with shells containing magnetite nanoparticles in capturing and transporting target substances was shown by Elkeles et al.^16^ Their team used a magnetic field to control the movements of the particles. Still, the release of the encapsulated substance was induced by pH changes. A peculiar example of magnetic nanocapsules is metal iron-based magnetoplasmonic drug-loaded nanocapsules.^17^ They contained a drug (paclitaxel) inside a polylactic-co-glycolic acid nanoparticle coated by Fe and SiO_2_ layers. The presence of Fe made it possible to magnetically manipulate the nanocapsules, but the release of the drug was triggered and depended on hydrolysis processes that take days to be completed. Magnetic core-shell nanocapsules with dual-targeting capabilities for drug delivery were described by Fang et al.^18^ They were used for brain glioma treatment in mice exhibiting high accumulation in the tumor, however, the drug release was controlled by the shell charges and pH regulation, rather than magnetic field. Hu et al^19^ demonstrated magnetic polyelectrolyte microcapsules for drug delivery containing MNs in the shell structure. The nanoparticles under a magnetic stimulus enabled the evolution of the shell structure from a nanocavity to the final rupture. While they successfully showed that the magnetic-field-sensitive microcapsules enabled quick absorption of the drug by the cancer cell line, only the release controlled by the magnetic field was evidenced (not navigation). Biodegradable polyelectrolyte/magnetite capsules for magnetic resonance imaging (MRI) and magnetic targeting of tumors were obtained by Svenskaya et al.^20^ MNs were placed either in the capsule shell or both in the shell and in the inner volume. An external magnet was applied for one hour after the capsules were intravenously injected. MRI scans acquired after administration of the capsules showed their significant accumulation in the tumor tissue and not in the other organs, but the capsules were considered as MRI contrast agents rather than systems for targeted delivery and controlled release of drugs.

Magneto-mechanical actuation is a phenomenon that has been established in several works as a potentially beneficial mechanism in various biomedical applications. It is one of the reasons why iron oxide nanoparticles are also excellent candidates for cell therapy, as shown by Beltran-Huarac et al.^21^ Their team managed to remotely control how the therapeutic TRAIL proteins in transduced cells were expressed using magneto-mechanical actuation of MNs with anisotropic shape and coated with nitrodopamine polyethylene glycol. Taking advantage of the properties of both magnetite and gold in bifunctional magnetite-gold nanoparticles may lead to enhancement of the magneto-mechanical actuation and cancer cell destruction, as shown by Garanina et al.^22^ Goršak et al^23^ employed a less typical shape of the nanoparticles, ferrimagnetic hexaferrite nanoplatelets to reduce the concentration of nanoparticles necessary to observe the magneto-mechanical action. Hillion et al^24^ closely investigated this process inside cells. They observed permeabilization of the cell membrane that happened a few minutes after the application of the magnetic field started. The MNs they studied generated mechanical forces that were strong enough to disrupt the lysosome integrity, as well as the leakage of the lysosome content. This effect occurred in response to the low-frequency rotating magnetic field. Kanber et al^25^ performed experiments on cells showing that magneto-mechanical actuation remotely induces endothelial permeability in a controlled manner, enabling exogenous substances to cross the endothelium in order to perform treatment of diseases such as cancer and others. In the work of Lunov et al^26^ it is demonstrated that it is possible to remotely induce apoptosis in liver cancer cells by magneto-mechanically modulating iron oxide nanoparticles.

Another phenomenon related to the therapeutic applications of MNs is magnetic hyperthermia. This process occurs when MNs are subjected to an alternating magnetic field and, as a result, generate heat in the targeted regions such as cancerous tissues.^27^ Hyperthermia increases the sensitivity of cancer cells to chemotherapy^28^ and can even induce death of cancer cells as they are more prone than normal cells to damage caused by elevated temperature.^29^ Application of the alternating magnetic field to MNs forces their moments to follow the magnetic field and triggers two relaxation mechanisms. According to the Néel mechanism, the magnetic moment of the nanoparticle can flip, while in the Brownian mechanism rotation of the whole particles occurs. Although those relaxation mechanisms may be hard to separate (they can occur simultaneously), the heating induced by the alternating magnetic field is attributed mainly to the Néel mechanism. Importantly, the Brownian mechanism usually dominates for the magnetic core size above 16 nm, while for the smaller nanoparticles (<12.5 nm) the Néel mechanism is dominant.^30^ The efficiency of magnetic hyperthermia treatments can be boosted by taking advantage of resonant spin-excitation and dissipation.^31^ It is desired to obtain smart, stimuli-responsive magnetic systems for drug delivery,^32^,^33^ but combining large magnetic moment, high loading capacity, and low cytotoxicity of the carriers is still a challenge.^34^

We have introduced polysaccharides-based capsules templated on oil cores that can efficiently carry hydrophobic active compounds and form a stable dispersion in aqueous media.^35–38^ Only recently, such polymer capsules with dispersed MNs and chitosan-based shells have been reported to serve as water dispersible nanoreactors.^39^,^40^ Such structures enable the encapsulation of hydrophobic compounds and magnetic control of the whole system.

In this work, we present a novel approach related to the controlled delivery and release of hydrophobic drugs based on magnetic core-shell nanocapsules. One of the distinctive features of such carriers is that magnetic field can be used for both their navigation and pulling into the interior of tumor cells (static field), as well as for an on-demand release (alternating field) of the carried hydrophobic payload.

The aim of this study was to develop nanocapsules as controllable nanosystem containing encapsulated magnetic nanoparticles and hydrophobic cargo molecules that can be navigated to selected cells using a static magnetic field and are capable of on-demand release of the encapsulated cargo upon exposure to alternating magnetic field stimuli.

## Materials and Methods

### Materials

Synthesis of N-[(2-hydroxy-3-trimethylamine) propyl] chitosan chloride (CChit) derivative (Figure S1) and further modification with dodecyl groups leading to amphiphilic cationic chitosan (CChit-C12) were carried out following the procedure described by Karewicz et al^41^ with modification and characterization reported earlier by us^39^ (details are given in SI file, section “Synthesis and characterization of cationic derivative of chitosan (CChit”). Synthesis of anionic N-sulfate derivative of chitosan (ACh) (Figure S2) was carried out using the modified procedure described by Bulwan et al^42^ with utilization of carboxymethyl chitosan (CMC, deacetylation degree 90%, AK Scientific, Union City, CA, US), sulfur trioxide-trimethylamine (TMST, 99%, Sigma-Aldrich, Saint Louis, MO, USA), sodium hydrogencarbonate (NaHCO_3_, analytical grade, Sigma-Aldrich), sodium hydroxide (NaOH, analytical grade, Avantor Performance Materials Poland S.A., Gliwice, Poland) and cellulose dialysis tubes (14,000 g/mol cut-off, Sigma Aldrich). The synthesis and characterization of ACh is described in the Supplementary Information (section “Synthesis and characterization of anionic derivative of chitosan (Ach”). FT-IR and ^1^H NMR spectra of ACh are presented in Figures S3 and S4, respectively. Hydrodynamic diameters (measured by DLS) of the anionic capsules after 48 weeks of storage are demonstrated in Figure S5 indicating high stability of the capsules and their homogeneous size.

For cationic and anionic capsules preparation oleic acid (OA, 99.5%, Alfa Aesar, Haverhill, MA, USA) was used. For confocal microscopy measurements fluorescent dyes: perylene (Pe, gold label, 99.9% Sigma-Aldrich) or Nile Red (NE, for microscopy, Sigma-Aldrich) were used. Cellular studies were performed using murine mammary gland cancer cell line (4T1, ATCC CRL-2539), Dulbecco Modified Eagle Medium (DMEM, High Glucose, Sigma-Aldrich), and Fetal Bovine Serum (FBS, HyClone™ Research Grade Fetal Bovine Serum, Thermo Fisher Scientific Inc., Waltham, MA, USA). Cell viability was determined by Cell Proliferation Kit II (XTT, Sigma-Aldrich), fluorescent dye Hoechst (Sigma-Aldrich), and Propidium Iodide (PI, Sigma-Aldrich). During the experiments, phosphate-buffered saline (PBS, tablets, Sigma-Aldrich), trypsin (HyClone), penicillin-streptomycin solution (Symbios, Gdańsk, Poland) ethanol (EtOH, 70%, Chempur, Piekary Śląskie, Poland), dimethyl sulfoxide (DMSO, ≥99,7%, Sigma-Aldrich), formalin (water solution 36.5–38%, Sigma-Aldrich), sodium chloride (NaCl, analytical grade, Sigma-Aldrich), acetone (analytical grade, Chempur) and deionized water were used.

### Preparation of the Cationic and Anionic Capsules with Superparamagnetic Iron Oxides Nanoparticles

Synthesis and characterization of the superparamagnetic iron oxides nanoparticles (SPION) coated by oleic acid as well as preparation of cationic chitosan-based oil-core nanocapsules with encapsulated SPION were reported by us earlier.^39^ Shortly, the preparation of the capsules is based on the emulsification process with self-assembling of the amphiphilic chitosan derivate on the dispersed oleic acid cores with suspended SPION. For this purpose, 10 μL of oleic phase with suspended SPION (ca. 100 g/L) was added to the 1 mL solution of the chitosan derivative (CChit-C12) dissolved in 0.15 NaCl (CChit-C12 concentration 1 g/L). The emulsification was achieved by intensive shaking (for 10 min) and pulse ultrasonication (for 30 min, 1s on/2s off mode) at room temperature.

Anionic capsules were obtained with the layer-by-layer method by mixing 0.4 mL dispersion of the previously prepared cationic capsules with 0.6 mL of the ACh (1 g/L) dissolved in 0.0015 M NaCl. Concentrations of ACh and NaCl were determined in optimization procedures. The resultant solution was intensely shaken for 10 min at room temperature.

### Preparation of the Capsules Containing Fluorescent Dyes

The procedure of the formation of both cationic and anionic capsules was modified by the addition of a proper fluorescent dye: perylene (1 mg/mL), Nile Red (5 mg/mL), to the oil suspension of SPIONs, which later formed the cores of the capsules.

### Cell Culture Preparation

4T1 line cell culture was grown using a medium (DMEM, High Glucose, Sigma-Aldrich) supplemented with an antibiotic in the form of penicillin and streptomycin solution (1%) and fetal bovine serum (5%). All reagents used in the cell studies were warmed up in a water bath at 37°C. In the first step, cells were thawed, the cell suspension was transferred to a centrifuge tube, and 5 mL of medium was added to dilute the dimethylsulfoxide (DMSO). Then, the cells were centrifuged (1000 rpm, 5 min), decanted from the solution above the cell pallet, and resuspended in 1 mL of medium. The cells were seeded on a dish to which 10 mL of the medium was previously added. The proliferation of cells and their condition during cultivation were monitored by an optical microscope, and the medium was changed every 2 days. Then, to perform the cell passage, trypsinization was carried out. The dish with the cells was rinsed with a PBS solution; the procedure was repeated two times. Subsequently, 0.8 mL of trypsin solution was added, drawn off, and again 0.8 mL of trypsin was added. The dish prepared was placed in the incubator; after 3 minutes it was tapped thoroughly and put back into the incubator. After another 3 minutes, the dish was tapped once more, and the degree of detachment of the cells from the substrate was monitored under the microscope. The detached cells were transferred from the dish to the Falcon tube with the prior addition of 3 mL of medium to inactivate the trypsin effect. The cell suspension obtained was centrifuged (1000 rpm, 5 minutes) and the cells were suspended in 1 mL of medium.

### XTT Assay

XTT assays were performed according to the manufacturer’s instructions with some modifications. Namely, two 24-well plates containing cells of the 4T1 cell line and the dispersions of cationic capsules (plate 1) and anionic capsules (plate 2) of the appropriate concentrations were prepared the day before the XTT assay. For each concentration of the capsules, the XTT tests were repeated three times. The plates were prepared by adding 0.9 mL of medium to each well and then adding 100 μL of cell suspension. Each well was mixed thoroughly by pipetting and shaking the plate several times. The seeded plates were placed in the incubator for 4 hours. The next step was to add the agent in the form of capsules. Fluid from each well was withdrawn and 0.9 mL of the medium was added. Then, 0.1 mL of capsules’ dispersion diluted (from 100, 50, 25, 17, 12,5, 10 times) in 0.15 M NaCl (for cationic capsules) or 0.0015 M NaCl (for anionic capsules) was added to each well, thus varying the concentration of the capsules in the range 0.01–0.1 µL/mL (cationic) and 0.004–0.04 µL/mL (anionic). The solutions obtained were mixed in the wells by pipetting. The plates prepared were placed in an incubator for 24 hours. To carry out the assay, the necessary reagents for the XTT test were prepared. According to the manufacturer’s instructions, solutions were warmed up to 37°C and then 5 mL of XTT labelling reagent and 0.1 mL of the coupling reagent were mixed together. Before adding the XTT mixture, the plates prepared the day before were removed from the incubator and the liquid in them was pipetted. About 0.2 mL of the new medium and then 0.1 mL of XTT solution was added to each well. The contents of the plates were gently mixed, and the plates were placed in the incubator. After 1 hour, the spectrophotometric measurement of the absorption was performed at the wavelengths of 460 nm and 700 nm. Figure S6 (in the SI file) shows the viability of 4T1 cells determined using XTT test in contact with cationic and anionic magnetic capsules.

### Navigation of the Capsules to the Cells Using Static Magnetic Field

**Figure 1:**
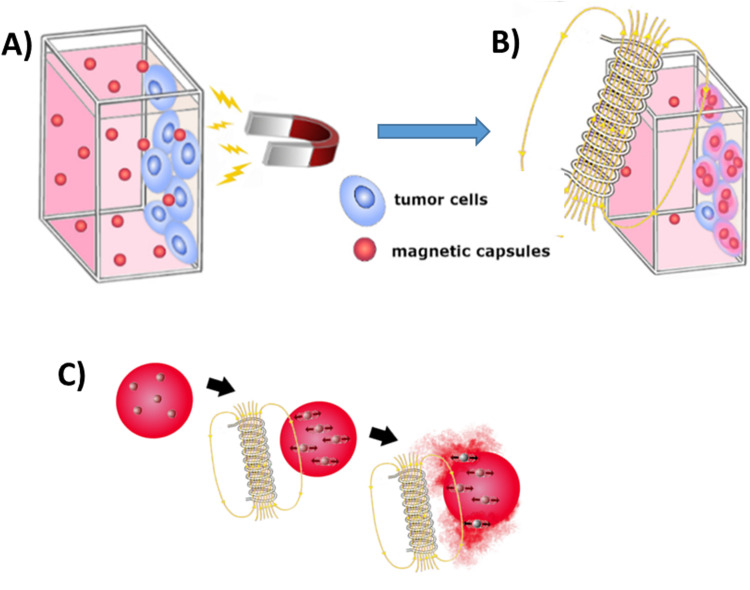
Scheme of the experimental setup for dragging the capsules into the cells using static magnetic field (**A**) and, as a continuation of the experiment, application of an alternating magnetic field to the cells (**B**); scheme of the mechanism of the capsules’ cargo release (**C**).

Experiments with static magnetic field were carried out in a specially designed system, which ensured no influence of the gravitational forces on the penetration of capsules into the cells. For this purpose, a microscopic coverslip covered by cells was vertically placed into a glass cuvette filled with capsules suspended in a medium and containing encapsulated Nile Red. Properly cut and sterile microscopic glass coverslips were used and placed in the 6-well cell plates where the cell culture preparation procedure, described above, was carried out. Then, the glass substrate with the cells was placed on the sidewall in a sterile cuvette. About 0.9 mL of the cell culture medium and 0.1 mL of the capsules dispersion diluted 50 times with 0.15 M NaCl (for cationic capsules) and 0.0015 M NaCl (for anionic capsules) were poured into the cuvette. The final concentration of the capsules used in these experiments was estimated (based on the oil content) to be equal to 0.020 µL/mL (cationic) and 0.008 µL/mL (anionic). The procedure of the experiment is illustrated in Figure 1A.

Such prepared systems were directly exposed for 5 min and 15 min to a static magnetic field acting perpendicular to the gravitation force (and to the side wall of the cuvette). The source of the static magnetic field was a neodymium magnet. The distance between the sample and the neodymium magnet was 1 cm, which corresponded to a magnetic field induction of 143 mT, and the gradient was 40 T/m. The specific magnetization of the nanoparticles at the field of this magnitude was equal to 12 emu/g,^3^ therefore the magnetic force acting on the nanoparticles was ca. 50 times greater than the gravitational force.

Then, the cells on the slide were fixed, which enabled their subsequent imaging using confocal fluorescence microscopy. For fixation, the cell slides were transferred to a 6-well plate and washed twice with 2 mL of PBS solution. Then, 2 mL of a 10% formaldehyde solution in PBS was added to each well. After 10 min, the solution was withdrawn, the slides were rinsed with PBS, and placed on a microscopic glass slide, protecting the edges with varnish.

### Release of the Encapsulated Substances Inside the Tumor Cells Using an Alternating Magnetic Field

Utilization of an external alternating magnetic field was the subsequent stage of the experiment. The sample system described in the previous section, after being subjected to a 15-minute exposure to a static magnetic field, was transferred for 5 min to the alternating magnetic field. Figure 1B illustrates the alternating magnetic field experiment procedure. The release mechanism is presented in Figure 1C. A 50 hz alternating magnetic field was produced by a transformer coil system with a vertical gap in its core of 20 mm width and an area of 25 cm^2^, where the sample was placed. The experiment was carried out at field induction values of 0–220 mT RMS (alternating between −310 and +310 mT). The samples were field treated for 5 min at magnetic induction equal to 22 mT (weak field), 67 mT (medium field), and 220 mT (strong field). The magnetic field was oriented horizontally, and its inhomogeneity within the sample volume was smaller than 15%. After the experiment, the slides with cells were fixed as it is described in the previous section (Navigation of the capsules to the cells using static magnetic field).

### Hoechst and Propidium Iodide Cell Viability Verification

Two solutions were prepared, 10 mg of Hoechst dissolved in 1 mL of deionized water and diluted with PBS solution in a volume ratio of 1:2000 and 1 mg of propidium iodide dissolved in 1 mL of deionized water and diluted in PBS in a ratio of 1:3000 by volume. Then, after the experiment with an external magnetic field, the cells on the glass slide were fixed with a suitable dye for 5 min, washed 3 times with PBS solution, placed on a microscopic glass slide, and protected with varnish. The samples obtained were imaged with a confocal microscope.

### Methods

NMR spectrometer (Avance III HD 400 MHz, Bruker, Billerica, MA, USA) equipped with gradient probes, operating in the normal mode and reverse detection was used to measure ^1^H NMR spectra. Elemental analysis was carried out using the Vario Micro Cube CHNS Analyzer by Elementar. The FTIR spectrometer (ALPHA FT-IR, Bruker) working in the attenuated total reflection (ATR) mode with a diamond attachment (for each sample, at least 128 scans were performed with a resolution of 4 cm^−1^) was applied to measure the FTIR spectra. Measurements of hydrodynamic diameters and zeta potentials were made using Nano ZS AP Instruments (Malvern Panalytical, Malvern, UK) device using a laser with a wavelength of 633 nm, the detection angle of 173° and at 22°C (the reported values were obtained from the average of three measurement series consisting of at least 10 repetitions each). For such dynamic light scattering (DLS) measurements, the original capsules’ dispersions were diluted 100 times. The cryogenic transmission electron microscope (cryo-TEM) imaging was performed using a TECNAI F20 TWIN microscope (FEI Company, Hillsboro, OR, USA). Fluorescence microscopy imaging was performed using a Ti-E inverted confocal fluorescence microscope (Nikon, Amstelveen, The Netherlands) equipped with an immersion objective Plan Apo 100x/1.4 Oil DIC and Nikon A1 confocal system equipped with lasers (405 nm and 561 nm) and DAPI (emission 425–475 nm) and TRITC (emission 570–620 nm) filter cubes. The absorption measurements were made using an Epoch 2 (BioTek Instruments, Winooski, VT, USA) spectrophotometric plate reader. The polymers were lyophilized using the Alpha 1–2 LD Plus (Christ, Osterode, Germany) freeze dryer. In the process of producing the capsules, a Vortex Genius 3 shaker (IKA, Warsaw, Poland) and a Sonic-6 ultrasonic bath with a power of 540 W (Polsonic, Warsaw, Poland) were used. The Nikon Eclipse TS 100 optical microscope was used for the imaging of cells. Magnetic induction of the neodymium magnet and the transformer coil system was determined using a GM08 gaussmeter (Hirst Magnetic Instruments Ltd. Tesla House, Tregoniggie, Falmouth, Cornwall, UK). If necessary for statistical significance of the results, the performed experiments were conducted at least three times.

## Results and Discussion

**Figure 2:**
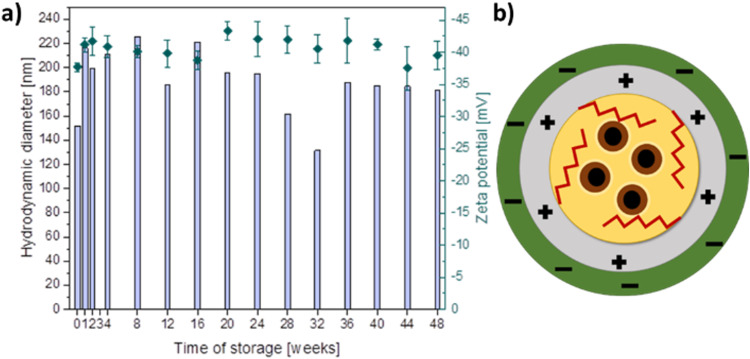
(**a**) Hydrodynamic diameters (bars, left scale) and zeta potential (points, right scale) values of anionic capsules with encapsulated SPION over 48 weeks of storage as determined with DLS; (**b**) scheme of the capsule with the chitosan-based shell (CChit-C12 cationic stabilizing layer and ACh anionic outermost layer) and SPION embedded in the oil core.

Magnetic core-shell capsules containing a dispersion of superparamagnetic iron oxide nanoparticles (SPION) in their oleic acid cores, stabilized by the cationic amphiphilic derivative of chitosan (CChit-C12)^41^,^42^ and then coated by anionic chitosan derivative (ACh)^42^ were obtained and characterized (see Supplementary Information (SI) for experimental details). The average diameters of the encapsulated SPION were estimated to be of 15 nm, as reported previously.^39^ The average hydrodynamic diameters of the anionic capsules (see Figure 2) were determined by DLS measurements to be ca. 200 nm and stayed in the range of 140–230 nm (with PDI below 0.4) during prolonged storage up to 48 weeks (examples of DLS data are presented in Figure S5). The zeta potential values also did not vary significantly and stayed in the range of −35 to −45 mV for almost a year of storage at 4°C. Earlier reports on cationic chitosan-based capsules^36^ indicated the average size of the hydrodynamic diameter in the range of 140–170 nm (with PDI below 0.3) and the zeta potential values from +30 to +40 mV. The obtained results confirmed high stability of the prepared formulations. Larger mean values of their hydrodynamic diameters in comparison with cationic capsules, as well as negative zeta potential values, indicate proper deposition of the anionic layer on the surface of the carriers.

**Figure 3:**
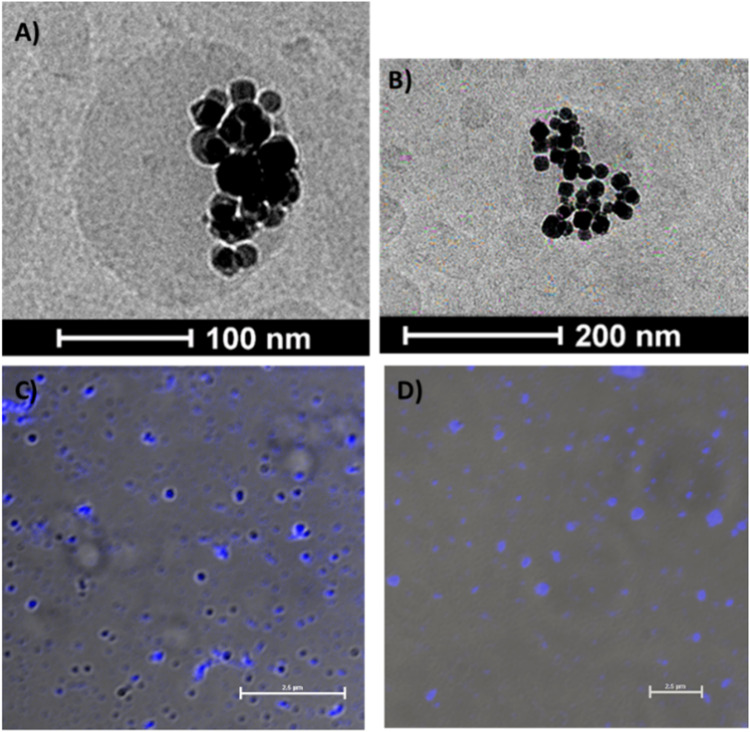
Cryo-TEM images of cationic capsules containing encapsulated SPION (**A** and **B**); Confocal fluorescence microscopy images of: cationic capsules (**C**), anionic capsules (**D**) with encapsulated perylene. The images were captured in a transmission light mode (λ_ex_ = 405 nm; TRICT filter; scale bars - 2.5 μm).

Cationic magnetic capsules were imaged using cryo-TEM (Figure 3A and B). The images obtained confirm the presence of SPION inside the nanocapsules, which are spherical in shape with diameters comparable to the ones determined from DLS measurements.

The capsules with encapsulated perylene, a hydrophobic fluorescent dye, were imaged using confocal fluorescence microscopy (Figure 3C and D). The images show a blue luminescence of perylene confirming its efficient encapsulation in the oil cores of the capsules since perylene can only fluoresce in a hydrophobic medium.

The tests of the cell viability in contact with the anionic or cationic capsules at various concentrations were performed. The originally obtained dispersions of the nanocapsules were diluted with 0.15 M NaCl (cationic capsules) and 0.0015 M NaCl (anionic capsules) 10 to 100 times. The results presented in Figure S6 show decreasing cells viability for the increasing concentration of the capsule. However, cationic capsules now showed a negative effect on the cell viability at concentration of ca. 0.02 µL/mL (50-fold dilution in Figure S6) that corresponds also to the concentration of SPION equal to 2 µg/mL. Thus, this concentration was selected for the magnetic navigation experiments. Similarly, for anionic capsules, the 50-fold diluted system was chosen with a concentration of the capsules equal to 0.008 µL/mL. Nevertheless, even much higher concentration of the capsules would be acceptable for the experiments based on the results of the XTT tests.

**Figure 4:**
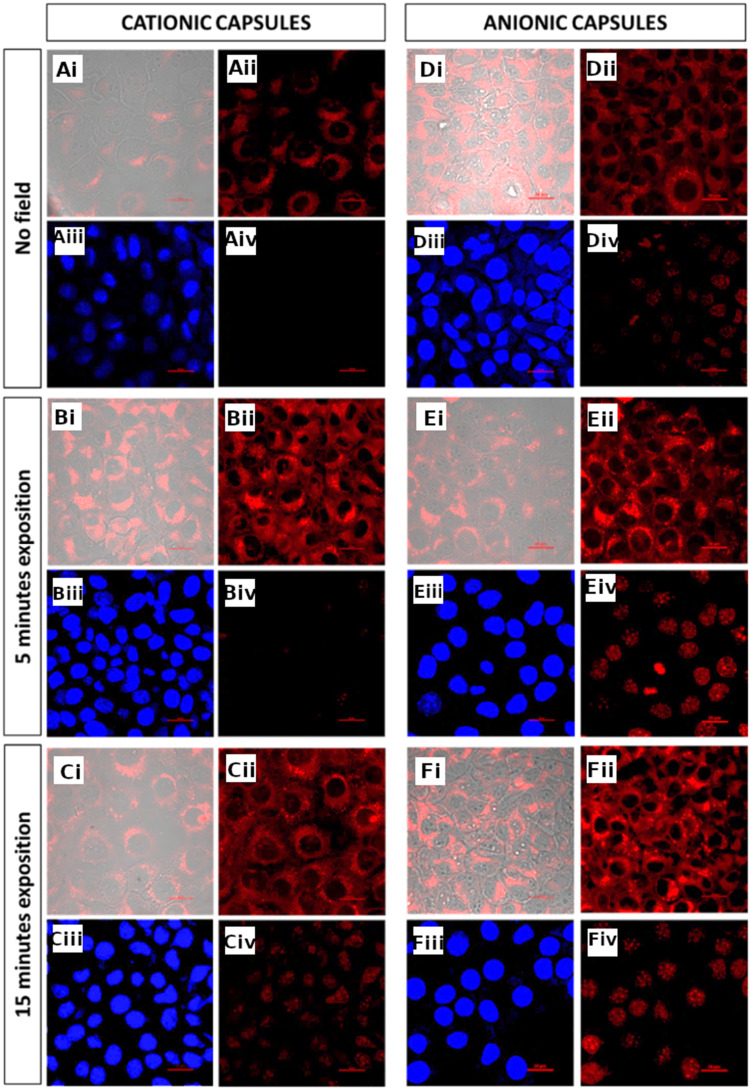
Confocal fluorescence microscopy images of cells at no magnetic field and after static magnetic field navigation of cationic or anionic capsules. Cationic capsules with no field (**A**), 5 min (**B**) and 15 min (**C**) exposition to magnetic field. Anionic capsules with no field (**D**), 5 min (**E**) and 15 min (**F**) exposition to magnetic field. Fluorescently labeled capsules, transmitted light and TRITC filter (i); fluorescently labeled capsules, TRICT filter (ii); cells stained with Hoechst, DAPI filter (iii); cells stained with propidium iodide, TRICT filter (iv); scalebars: 20 μm.

Confocal microscopy images of the cells incubated for 5 and 15 min with cationic and anionic capsules with and without the application of a magnetic field are presented in Figure 4. The magnetic nanocapsules with encapsulated model hydrophobic dye (Nile Red) were subjected to a static magnetic field in order to move them towards the cells deposited on a glass substrate. Images of the cells incubated with cationic capsules that were not exposed to a magnetic field indicate no spontaneous penetration of the cancer cells by the carriers (Figure 4 images Ai-Aii), whereas capsules with negative surface charge seem to be able to overcome the biological membrane of the cells by endocytosis (Figure 4 images Di-Dii). Utilization of the static magnetic field enabled internalization of positively charged capsules (Figure 4 images Bi-Bii, Ci-Cii) and enhanced uptake of the anionic capsules (Figure 4 images Ei-Eii, Fi-Fii).

Those observations were supported by the images of the cells stained by Hoechst dye (blue emission, membrane-permeable dye; stains DNA) and propidium iodide (red emission, membrane impermeable dye; stains DNA and indicates loss of the membrane integrity) presented in Figure 4 (subfigures Aiii-Aiv, Biii-Biv, Ciii-Civ, Diii-Div, Eiii-Eiv, Fiii-Fiv). The absence of red fluorescence in Figure 4 Aiv and its presence in Figure 4 Div reveals the presence of spontaneous intracellular uptake of anionic capsules. Nevertheless, the magnetic dragging of the nanocapsules towards the cells significantly increases the effectiveness of their incorporation and leads to some cell membrane damage, especially after a longer exposure to the magnetic field (Figure 4, subfigures Biii-Biv, Ciii-Civ, Eiii-Eiv, Fiii-Fiv). The overall intensities of the indicative red fluorescence in Figure 4 Aiv, Biv, Civ (cationic capsules) and Figure 4 Div, Eiv, Fiv (anionic capsules), normalized to a single cell, were calculated and presented for comparison in Table S1 showing a clear growing trend with the time of applied magnetic field. Thus, the enhancement of the uptake of the nanocapsules can be attributed to magnetically navigated forced delivery of the capsules. Importantly, significantly more efficient internalization of anionic capsules (compare Figure 4 images Biv and Eiv) was observed in spite of their 60% lower concentration as compared to cationic capsules. It also indicates the importance of the capsules’ shell properties on the efficiency of their forced intracellular delivery. Control experiments showed no negative impact of the applied magnetic field on the permeability of the membranes of the cancer cells in the absence of the capsules (Figure S7).

After the efficient incorporation of magnetic nanocapsules into the cancer cells induced by a static magnetic field, the next step was the controlled release of the cargo substance by an appropriate external alternating magnetic field. The tested cells were loaded with the nanocapsules by 15 min exposure to a static magnetic field. The possible negative influence of an alternating magnetic field on the cells had to be priorly ruled out in a control experiment. The images obtained confirm the preserved integrity of the cell membrane after their exposure to alternating magnetic field, which is indicated by the lack of fluorescence of cells stained with propidium iodide (Figure S8).

**Figure 5:**
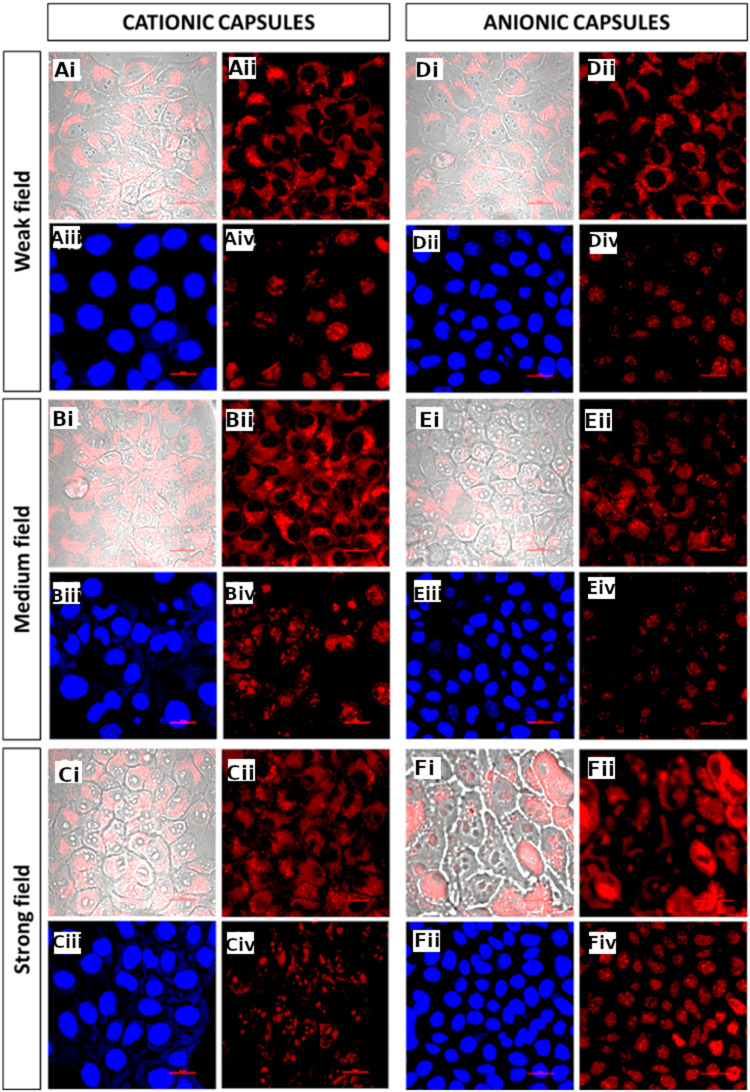
Confocal fluorescence microscopy images of cells loaded with nanocapsules using static magnetic field and then exposed to alternating magnetic field. Cationic capsules exposed to weak (**A**), medium (**B**), and strong alternating magnetic field (**C**); anionic capsules exposed to weak (**D**), medium (**E**), and strong alternating magnetic field (**F**). Fluorescently labeled capsules, transmitted light and TRITC filter (i); fluorescently labeled capsules, TRICT filter (ii); cells stained with Hoechst, DAPI filter (iii); cells stained with propidium iodide, TRICT filter (iv); scalebars: 20 μm.

The obtained images (Figure 5 images Ai-Aii, Bi-Bii, Ci-Cii) show that for the cationic capsules with encapsulated fluorescent dye, application of an alternating magnetic field seems to maintain the forced membrane permeation effect (compare Figure 4 images Ci-Cii and Figure 5 images Bi-Bii, Ci-Cii), but does not lead to a significant release of the encapsulated substance inside the cell.

An analogous experiment conducted for capsules with negative surface charge similarly indicates the support of their magnetic field-assisted penetration of cancer cells by the application of alternating magnetic field of weak and medium intensity (Figure 5 images Di-Dii, Ei-Eii). Furthermore, the results obtained for the capsules exposed to a strong alternating magnetic field clearly show the release of the encapsulated dye inside the tumor cells, which become fully filled with red fluorescence (Figure 5 images Fi-Fii). The images look similar to the ones obtained for hyaluronic acid-based nanocapsules of similar structure that were internalized by the cell spontaneously followed by the release of the cargo molecules due to the action of intracellular hyaluronidase enzyme destroying the shells of the capsules.^34^

The violation of the cell membrane integrity is revealed in the images of fluorescently stained cells after the experiment of their exposure to a static magnetic field and then to the alternating magnetic field (Figure 5 images Aiii-Aiv, Biii-Biv, Ciii-Civ, Diii-Div). The captured images indicate the possibility of introducing the capsules inside the cells with the disruption of the cell membrane that may lead to cell death. The intense red fluorescence spanning over the whole cells indicates significant damage to the cell membrane caused by the action of anionic capsules, especially in the case of application of the strong alternating magnetic field (Figure 5 images Fi-Fiv). These damages concern all the cells captured in the field of view of the image and ultimately lead to the death of cancer cells. Additional confocal fluorescence microscopy images excluding the negative influence of the static and alternating magnetic field are shown in Figures S7 and S8 (SI file).

## Conclusion

In summary, magnetic core-shell nanocapsules containing SPION in the oil core and chitosan-based shells were shown to be efficiently navigated into selected cancer cells by applying a directional static magnetic field and then, upon application of an alternating magnetic field, they released the cargo molecules. A proper design of the carriers enabled forced delivery of actives to the cells without the necessity of employing specific ligand-receptor interactions breaking the limitations of typical endocytosis. To the best of our knowledge, such carriers of hydrophobic drugs, combining forced, targeting-free delivery and on-demand intracellular release of the cargo molecules, controlled solely by a magnetic field as an easily applicable stimuli, are shown for the first time. Owing to the application of the chitosan-based amphiphilic polymer, the capsules formed are found to be non-cytotoxic at relevant concentrations, especially if coated with anionic derivative of chitosan. Such negatively charged capsules exhibit both the largest intracellular uptake, as well as an efficient release of the cargo molecules at a proper intensity of an alternating magnetic field, more effective than for positively charged carriers. The systems that we propose are versatile and can be further developed by varying the encapsulated drugs, magnetic nanoparticles, as well as the thickness of the formed shell implying the desired size, stability, and sensitivity of the carriers to the applied magnetic field.

## Abbreviations

MNs, magnetic nanoparticles; CChit, N-[(2-hydroxy-3-trimethylamine) propyl] chitosan chloride; CChit-C12, amphiphilic cationic chitosan with grafted dodecyl chains; ACh, anionic N-sulfate derivative of chitosan.
